# Axillary Recurrence in Breast Cancer Patients After Negative Sentinel Lymph Node Biopsy: Retrospective Cohort Study From Riyadh, Saudi Arabia

**DOI:** 10.7759/cureus.20132

**Published:** 2021-12-03

**Authors:** Roqayah I Alsunitan, Abdulaziz Al-Saif, Bader A Alyousef, Saud M Alghamdi, Shaimaa A Bugshan

**Affiliations:** 1 General Surgery/Breast and Endocrine Surgery, King Khalid University Hospital, Riyadh, SAU; 2 General Surgery/Breast and Endocrine Surgery, King Saud University Medical City, Riyadh, SAU; 3 General Surgery, King Khalid University Hospital, Riyadh, SAU; 4 Medicine and Surgery, King Khalid University Hospital, Riyadh, SAU

**Keywords:** sentinel lymph node, minimally invasive, breast cancer, biopsy, axillary lymph node dissection

## Abstract

Breast cancer (BC) is among the most prevalent cancers globally. For minimally invasive axillary staging in early breast cancer, sentinel lymph node biopsy (SLNB) is commonly regarded as the gold standard. Historically, axillary lymph node dissection (ALND) was used as a staging procedure, but the less morbid SLNB has now replaced it. This retrospective cohort study, undertaken with patients at King Saud University Medical City in Riyadh, Saudi Arabia, evaluates mid-term follow-up data on axillary recurrences and outcomes for breast cancer patients with negative SLNB. The results indicate that the five-year risk of developing regional recurrence following negative SLNB is 0% in breast cancer patients. The low relapse rate further contributes to the evidence base suggesting the efficacy of SLNB and the higher morbidity associated with ALND. Future researchers should conduct a nationwide and long-term follow-up study to offer additional insights into the efficacy of SLNB.

## Introduction

Breast cancer (BC) is among the most prevalent cancers [1]. The age-standardized rate of BC in the Kingdom of Saudi Arabia (KSA) is 24.9 per 100,000, while at diagnosis, the median age is 49 years. Over 50% of BC patients in the KSA reportedly suffer from locoregional and distant disease [2]. Due to technological and procedural improvements in diagnostic imaging, cancer treatment, and timely detection, BC’s long-term survival rate has improved in recent years [3].

The method of sentinel lymph node biopsy (SLNB) was first reported in the context of BC in 1994 [4]. For axillary staging in early BC management, SLNB is regarded as the best available staging procedure. Compared to axillary lymph node dissection (ALND), it is associated with reduced arm and shoulder morbidity, and improved quality of life [5].

The accuracy and feasibility of SLNB have been investigated, but few studies have focused on the mid-term and long-term follow-up results. Hence, this retrospective study’s aim was to assess mid-term follow-up data regarding the axillary recurrences and outcomes of BC patients with negative SLNB in Saudi Arabia.

## Materials and methods

Study design and setting

A retrospective cohort study was undertaken at King Saud University Medical City in Riyadh, KSA. IRB was approved by King Saud University College of Medicine. IRB approval was given by King Saud University, College of Medicine and Science.

Participants

After applying inclusion/exclusion criteria, 16 female patients were included in the study who had early-stage breast cancer (T1-T3), clinically negative axilla, and SLNB between January 2015 and December 2015.

Inclusion criteria

Patients were included if they satisfied the following criteria: aged 18 or older; evaluated as T(1-3) N0 M0 regarding TNM stage; uni-centric or multi-centric BC; breast-conserving therapy or mastectomy; before and after neo-adjuvant; and provided written consent to participate.

Exclusion criteria

Patients were excluded if any of the following criteria were satisfied: pregnant or lactating women; received additional ALND for validation after negative SLNB; micro or macro metastases detected in sentinel lymph nodes; or positive lymph node metastases confirmed using biopsy.

Data collection procedure

Combined isotope and blue dye (Bleu Patente V, Sodique Guerbet 2.5%, France) were used for SLNB. All procedures were undertaken using general anesthesia at King Saud University Medical City. Radiotracer administration occurred in a timely fashion (i.e., morning for afternoon procedures, or late-afternoon for procedures scheduled the next morning). Blue dye administration occurred shortly after anesthetizing the patient. Vials contained 1.5 mL Tc99m sodium pertechnetate (GE Healthcare Limited, UK), with activity ranging from 5 to 100 mCi (185-3700 MBq). Four syringes were prepared from the vial, each containing 0.1-0.2 mL Tc99m albumin colloid with 0.2-0.4 mCi (7.4-14.4 MBq). For multi-focal and multi-centric tumors, syringes were injected either in the periareolar area or four peritumoral sites, after which the nurse massaged the breasts (lasting 30 minutes) to ensure the migration of particles toward the axilla. Fifteen minutes post-injection, lateral and anterior images were obtained using the Phillips SPECT-CT system (Andover, Massachusetts, USA); this procedure was repeated until the SN became visible or when the radiologist decided the result was negative. 2.5 mL sterile saline with 2.5 mL of blue dye were combined and injected sub-dermally around the tumor, followed by a five-minute breast massage. A lymph node was exclusively classified as SN when blue-stained or when a blue lymphatic channel was observed leading to it, or when it is a hot node, as determined using the gamma detector probe (GPS Navigator GPS-9100-00 Dynasil, USA). Following sentinel lymph node excision, residual radioactivity was tested for in the axillary basin.

Pathologic examination of lymph nodes

Lymph nodes classified as SN were sent for frozen section. At the frozen section laboratory, lymph nodes were sliced perpendicular to the long axis, touch preparation was submitted from the cut surfaces, and staining was undertaken with modified Giemsa stain (Diff-Quick). After slicing, lymph nodes were submitted for frozen section, and equidistant slicing (2.0 mm thickness) was applied. For doubtfully negative H&E cases, immunohistochemistry was applied. Macro metastases were classified as metastatic lesions exceeding 2.0 mm in diameter, while micro metastases ranged from 0.2 to 2.0 mm. ALND (level 1 and level 2) was performed immediately if the SN was positive for metastasis; otherwise, it was not performed. The proposed intervention was undertaken while waiting for the outcome of frozen section.

Post-operative follow-up

Diagnostic post-operative follow-up procedures included clinical breast and axillary lymph node examination every 4-month period, along with annual mammography. Breast ultrasound was used to verify suspicious mammographic results.

Statistical analysis

The Statistical Package for the Social Sciences (SPSS 22; IBM, New York, USA) was used for data analysis. Mean ± standard deviation was used to express continuous variables, while percentages were used for categorical variables. The level for statistical significance was P < 0.05.

## Results

All participants were Saudi females (n=16). The mean age of the participants was 59.3 ± 10.1 (range: 47-82 years), while the mean age at diagnosis was 54.0 ± 9.6 (range: 42-76 years). Weight, height, and BMI were not recorded for three participants. For the 13 participants whose BMI data were available, mean BMI was 32.3 ± 6.9 (range: 23.6-46.9). Table 1 summarizes the participants’ sociodemographic characteristics.

**Table 1:** Participants’ sociodemographic characteristics.

Characteristics		N	%
Gender	Female	16	100.00
Nationality	Saudi	16	100.00
Menopausal Status	Pre-menopausal	1	6.25
	Peri-menopausal	3	18.75
	Post-menopausal	12	75.00
Age (Mean ± SD)		59.31 ± 10.08	
Weight (Mean ± SD)		76.53 ± 15.70	
Height (Mean ± SD)		156.14 ± 6.96	
BMI (Mean ± SD)		32.32 ± 6.94	

Surgical interventions included unilateral mastectomy (n=8), bilateral mastectomy (n=1), and lumpectomy (n=7). All patients received SLNB, and a mean of 2.8 sentinel lymph nodes were harvested. Table 2 shows the full medical and surgical histories of the participants.

**Table 2:** Participants’ surgical and medical histories. ALND: axillary lymph node dissection; SLNB: sentinel lymph node biopsy.

		N	%
Family history	Negative	16	100.0
Cancer duration	< 2 years	16	100.0
Tumor type	Ductal	14	87.5
	Lobular	2	12.5
Tumor size	T1	10	62.5
	T2	5	31.3
	T3	1	6.3
Location	Left	11	68.8
	Right	5	31.3
Breast cancer stage	1.0	16	100.0
Receptors’ status	ER	+ve: 13 -ve:3	+ve: 81.3 -ve: 18.8
	PR	+ve: 11 -ve:5	+ve:68.8 -ve:31.3
	HER	+ve: 6 -ve:10	+ve:37.5 -ve 62.5
ALND	None	16	100.0
Surgical procedure type	Bilateral Mastectomy + SLNB	1	6.25
	Left Mastectomy + SLNB	6	37.5
	Right Mastectomy + SLNB	2	12.5
	Lumpectomy + SLNB	7	43.75

Table 3 shows the therapies patients received following surgical intervention. Over the five-year follow-up, no patients developed ipsilateral breast tumor recurrence (IBTR) according to mammography and clinical examination.

**Table 3:** Therapies administered to participants.

Chemotherapy		N	%
Received	Yes	7	43.8
	No	9	56.3
Number of cycles	6 cycles	4	25.0
	Unknown number of cycles	3	18.8
Radiation therapy		N	%
Radiation therapy	Yes	5	31.3
	No	11	68.8
Area treated	Breast	5	33.3
	No	10	66.7
Number of sessions	No	10	62.5
	Unknown	6	37.5
Dosage	No	10	62.5
	Unknown	6	37.5
Hormonal therapy		N	%
Received	Yes	14	87.5
	No	2	12.5
Type of hormonal therapy		N	%
Tamoxifen		6	37.5
Femra		4	25
Herceptin & Tamoxifen		1	12.5
Herceptin & Femra		1	6.25
Herceptin & Femra & Tamoxifen		1	6.25

## Discussion

Breast cancer

BC is the most prevalent malignancy experienced by females worldwide. To plan health measures effectively, BC incidence and mortality must be understood. The Global Cancer Observatory (GLOBOCAN) has reported that 25.1% of all malignancies in women are BC [6]. Between 2001 and 2008, the Saudi Cancer Registry (SCR) recorded 6,922 cases of BC in women, where the 30-44 and 45-59 age groups had the highest proportion of BC cases (38.6% and 31.2%, respectively). The highest BC prevalence rate in the KSA is in the Eastern Province (26.6 per 100,000), followed by Riyadh and Makkah (2.05 and 19.4, respectively) [2].

Knowledge of the molecular underpinnings of BC biology and pathogenesis has greatly increased in the past decade. For BC to develop, the evidence indicates that the activation or inactivation of specific genes plays a key role [7].

Taken together, the available data indicate that BC incidence has risen in recent years, but mortality rates have also fallen [8]. In the developed countries, BC incidence is greater compared to developing countries, but the mortality rate is higher in the latter. This is attributable to developments in screening programs, improvements in targeted therapy, and growing awareness about the criticality of self-breast examination (BSE) [6].

Ethnicity is a factor that may be associated with BC incidence and mortality rates. Although BC treatment and survival rates have recently improved substantially, ethnicity-related differences have still been identified. This has led some researchers to hypothesize that certain ethnic groups have greater genetic susceptibility to BC [9], but other researchers have stated that socioeconomic factors are the prime variables that account for between-group differences disparities in BC incidence and mortality rates [8].

Geographic disparities in BC incidence have been accounted for in some studies as being attributable to dietary choices and environmental factors [10]. This information has been provided by collective studies of migrants relocating from low-risk areas to high-risk areas [11]. Higher BC risk is associated with low-fiber diets, meat- and fat-rich eating habits, sedentarism, and high BMI [12]. Creating more educational opportunities for women worldwide, along with raising awareness regarding BC-specific information, have been identified as strategies for improving BC detection and management. Furthermore, health policymakers should pay close attention to BC control and prevention, and in developing countries, it is especially critical to raise awareness of risk factors and early detection.

Breast cancer treatment

BC treatment involves local disease treatment using surgical intervention, radiation therapy, chemotherapy, biologic therapy, and hormonal therapy, or a combination. BC treatment is influenced by several prognostic, predictive, and patient-specific considerations, including tumor histology, axillary lymph node status, patient age, comorbidities, menopausal status, BC metastasis, and clinical/pathologic characteristics of primary tumor [13].

Axillary lymph node dissection

ALND was introduced in the 1800s as a way to stage BC and facilitate local control. Since then, ALND has routinely been used to identify women with axillary nodal metastases. Recently, SLNB, replaced the use of ALND as a staging procedure [14,15]. In females without axillary nodal metastases, ALND use fell from 94% to 36% between 1998 and 2004, while 68% of patients diagnosed with sentinel node metastases received ALND in 2004 [16]. Although ALND enables effective regional control, it is strongly associated with complications such as numbness, lymphedema, axillary web syndrome, and reduced range of motion for upper extremities. In the 1990s, the American College of Surgeons Oncology Group (ACOSOG) performed a multicenter trial (Z0011) to investigate the influence of ALND on patients with a positive sentinel node [1e3]. Due to the insignificant findings, it was concluded that ALND can be safely avoided in patients with limited sentinel node metastatic BC who are undergoing systemic adjuvant therapy and breast conservation therapy [17]. As such, the ACOSOG trial recommended that surgeons should avoid ALND for patients who satisfy the trial’s eligibility criteria. In a recent ACOSOG Z0011 randomized trial, which included patients with clinical T1-2 N0 M0 BC and a positive SN to ALND or no further axillary surgery, no evidence was identified for a therapeutic benefit of ALND in patients with limited nodal illness [18].

Sentinel lymph node biopsy

The theoretical basis of SLNB is that the first axillary lymph node (or nodes) that the breast drains into should be excised. There are two techniques for SLNB: first, intra-operative introduction of blue dye into the breast parenchyma, after which - following a skin incision at the axilla - any blue nodes are removed; and second, the introduction of radioactive colloid matter into the breast, after which a gamma probe is used to identify and excise any radioactive lymph nodes [19]. SLNB is the current gold standard for axillary staging in early BC, and it is associated with reduced rates of locoregional recurrence, reduced arm and shoulder morbidity, and improved quality of life in comparison to ALND [16].

King Saud University Medical City in 2011 performed a retrospective review of a prospective database of BC patients at King Saud University Medical City and the Research Institute at King Saud University Medical City. After SLNB was harvested without ALND, four patients (0.26%) presented with localized axillary recurrences, 54 patients (3.53%) presented with localized recurrences in the ipsilateral breast and chest wall; and 24 patients (1.57%) presented with distant metastases [20]. Noteworthily, recurrence was not found in any of the patients in our study. Future research nationwide in the KSA is expected to offer a more precise measurement of post-SLNB recurrence in the country.

## Conclusions

This retrospective cohort study, involving 16 early-stage BC patients in Riyadh, Saudi Arabia who underwent SLNB in 2015 and received a negative result, was undertaken in order to examine axillary recurrence rates after five years. While ALND was for a long time the gold standard for BC staging, facilitating local control, and minimizing regional recurrence, recent studies, including the largescale multicenter ACOSOG trials, have recommended against the use of the procedure except in limited cases, and especially for patients with negative SLNB. Our results, in showing that BC patients with negative SLNB had a 0% five-year probability of regional recurrence, support the growing body of literature attesting to the safety and efficacy of this procedure in both staging and control. On the basis of these results, we recommend that SLNB should be applied in routine clinical practice as the standard of care for BC primary staging and control in patients with negative SLNB.
